# Drug-resistant bacterial infections: We need urgent action and investment that focus on the weakest link

**DOI:** 10.1371/journal.pbio.3001903

**Published:** 2022-11-16

**Authors:** Christiane Dolecek, Sadia Shakoor, Buddha Basnyat, Tochi Okwor, Benn Sartorius

**Affiliations:** 1 Centre for Tropical Medicine and Global Health, Nuffield Department of Medicine, University of Oxford, Oxford, United Kingdom; 2 Mahidol Oxford Tropical Medicine Research Unit, Mahidol University, Bangkok, Thailand; 3 Department of Pathology and Laboratory Medicine; Department of Pediatrics and Child Health, Aga Khan University and Hospitals, Karachi, Pakistan; 4 Oxford University Clinical Research Unit—Nepal, Kathmandu, Nepal; 5 Patan Academy of Health Sciences, Kathmandu, Nepal; 6 Nigeria Centre for Disease Control, Abuja, Nigeria

## Abstract

In a world of multiple crises, the momentum to tackle drug-resistant bacterial infections must not fall further behind. A renewed focus is needed on global infection prevention and control strategies, which are lagging behind in many countries, diagnosis and good antimicrobial stewardship.

## Teaser

Despite high mortality and morbidity, drug-resistant bacterial infections remain the forgotten pandemic. We argue for strengthening of diagnostics, WASH (water, sanitation, and hygiene) and infection prevention and control to reduce drug-resistant infections, as an integral part of sustainable high-quality health services, particularly in low- and middle-income countries.

Drug-resistant bacterial infections threaten the whole of modern medicine and public health, from the ability to conduct routine surgery, to prevention and treatment of infections compromising chemotherapy, to safe childbirth, treatment of sepsis, or management of tuberculosis.

In January 2022, we reported the most comprehensive estimates of the global burden of bacterial antimicrobial resistance (“the GRAM report”), estimating that antimicrobial resistance was a contributing factor in 4.95 million deaths in 2019; of those, 1.27 million deaths were directly attributable to antimicrobial resistance (AMR) [1,2]. The study highlighted inequities, with the all-age death rate attributable to AMR highest in sub-Saharan Africa (23.7/100,000), despite this region not having the highest AMR prevalence, but because of the high infectious disease burden and fragile health systems.

The GRAM report [1] has been widely cited, presented to G7 and G20 leaders, and discussed among global public health and clinical communities. The results provided more evidence to support the alarm that the World Health Organization (WHO) had been raising over the last decade—that drug-resistant infections are now a global health crisis that affect all countries, but pose the greatest burden on the most vulnerable—which led to the adoption of the political declaration on AMR by the UN General Assembly in 2016 [3].

On the occasion of World Antibiotic Awareness Week 2022 [4], we would like to reflect on progress and ongoing international efforts. Over the last year, we have seen dramatic changes in the geopolitical landscape. Russia’s full-scale invasion of Ukraine, extreme draught ravaging the Horn of Africa, devastating floods in Pakistan, the global energy and food crisis, inflation, and a severe downturn in the global economy. In addition, almost three years after it emerged, the world continues to be in the throes of the Coronavirus Disease 2019 (COVID-19) pandemic. As a result, the momentum and urgency to tackle AMR has faded into the background—yet the AMR crisis continues to worsen.

The main principles to combat bacterial drug-resistant infections have been clearly defined and agreed upon [5]: (1) reducing the number of infections; (2) the optimal use of existing antibiotics (including securing timely access to quality antibiotics when needed); and (3) the development of novel antibiotics, vaccines, and diagnostic tests; these actions must be accompanied by awareness campaigns, education and training, and AMR surveillance (see Box 1). However, their implementation has proven much harder. Most countries have developed AMR National Action Plans (NAPs), aligned with WHO’s Global Action Plan on AMR, outlining how they will tackle drug-resistant infections using a One Health approach that encompasses the human, animal, agricultural, and environmental sectors [6].

A lot remains to be done—even in wealthier countries, particularly in incentivising antibiotic stewardship, Research and Innovation (R&I), and supporting pull mechanisms to delink antibiotic development from sales volume [7]. However, the reality in many low- and middle-income countries is that dedicated resources for the actions necessary to deliver these plans are lacking, jeopardizing their implementation [8,9].

**Box 1. The five main objectives for combatting antimicrobial resistance**

**1. Reducing the number of infections**

Through vaccination, improved sanitation and clean water (WASH), and strengthening infection prevention and control (IPC) in health facilities and the community.

This is particularly important in low- and middle income countries, due to their higher overall infectious disease burden.

**2. Optimal use of antibiotics (antibiotic stewardship)**

Using appropriate antibiotics (according to WHO AWARE categories) in a timely fashion when needed, recognising the life-saving potential of these drugs, while at the same time reducing unnecessary use.

Availability and adherence to treatment guidelines.

**3. Research and innovation**

Development of new antibiotics, particularly new powerful classes of antibiotics.

Developing new bedside diagnostic tests to distinguish between viral and bacterial infections.

Developing and licensing of new vaccines against bacterial infections, particularly where no effective vaccine exists yet.

**4. Awareness**

Creating awareness of AMR among the general population.

Education and training of healthcare workers.

**5. Surveillance and data**

Strengthening the knowledge base and evidence about AMR through national and supranational surveillance systems.

Tracking of healthcare-associated infections (HAIs).

Box 1 adapted from the WHO Global Action Plan on AMR [5]

An essential component of the NAPs is Infection Prevention and Control (IPC) in healthcare facilities, i.e., measures directed at protecting patients and healthcare workers, and in consequence the community, from acquiring and being harmed by avoidable healthcare-associated infections (HAIs) and AMR [10]. Sadly, HAI remain neglected, particularly in low-resourced settings, predominantly affect vulnerable populations, and inflict high morbidity and mortality. As the GRAM report highlighted, the highest number of fatalities attributable to AMR are caused by six leading bacterial pathogens that are also important and prominent causes of HAI, with *Klebsiella pneumoniae* responsible for the highest attributable burden in sub-Saharan Africa and *Escherichia coli* in South Asia [1]. Adequate IPC is essential to the delivery of all modern medicine through high-quality health services. Hand hygiene in combination with environmental cleaning and antibiotic stewardship has been proven to be cost-effective and save lives, by reducing HAI and associated illnesses [10]. As the main pillar of IPC and its most essential intervention, hand hygiene needs to be applied in multimodal bundles in healthcare facilities that incorporate training and real-time feedback loops to be effective and support the necessary behavioural change [10]. Sadly, the dismal and shocking state of WASH services, especially in high AMR-burden settings, prevents implementation of IPC activities. An estimated 50% of health facilities in the 47 least-developed countries do not have basic water services [11]. The same issues apply to sanitation and waste management. This needs urgent attention and investments into the built infrastructure of health facilities, as well as the availability of basic materials such as soap and alcohol rub, training, and adequate staffing levels.

While the COVID-19 pandemic was a setback for IPC and AMR even in highly resourced regions due to reallocation of resources, shortage of healthcare workers and increased use of antibiotics [12], it has been devastating in low-income countries and has pushed back the AMR agenda manyfold in these settings.

The fight against AMR requires an intensified focus on strengthening health systems and health infrastructure globally, as well as putting sustainable pathogen surveillance and IPC at its centre. A horizontal approach will provide long-term benefits across the whole of modern medicine and public health, including for the detection, prevention, and response to epidemics and pandemics. To that end, a harmonized global push to advance WASH reforms across Asia, Africa, and the Global South is urgently needed.

The “AMR review” projected 10 Mio deaths annually in the year 2050 [13] attributable to AMR, which was criticised at the time. However, the GRAM report estimates emphasize that we are on a dire trend. We must urgently refocus on AMR; otherwise, more precious lives will be lost.

## Abbreviations

AMR: antimicrobial resistance
IPC: infection prevention and control
NAP: National Action Plan
R&I: Research and Innovation
WASH: water, sanitation, and hygiene
WHO: World Health Organization
